# Basal Cell Carcinoma of the Head and Neck Region in Ethnic Chinese

**DOI:** 10.1155/2011/890908

**Published:** 2011-09-14

**Authors:** Velda Ling Yu Chow, Jimmy Yu Wai Chan, Richie Chiu Lung Chan, Joseph Hon Ping Chung, William Ignace Wei

**Affiliations:** Division of Head and Neck, Plastic and Reconstructive Surgery, Department of Surgery, Queen Mary Hospital, The University of Hong Kong, Pokfulam Road, Hong Kong

## Abstract

*Objectives.* This study aims to report our experience in the management of HNBCC in ethnic Chinese over a 10-year period. *Methods.* A retrospective review of all ethnic Chinese patients with HNBCC treated in a tertiary centre from 1999 to 2009. *Results.* From 1999 to 2009, 225 patients underwent surgical excision for HNBCC. Majority were elderly female patients. Commonest presentation was a pigmented (76.2%) ulcer (64.8%) over the nose (31.6%). Median skin margin taken on tumour excision was 2.0 mm; primary skin closure was achieved in 51.8%. Postresection skin margin was clear in 75.4%. Of those with inadequate skin margins, 56.7% opted for further treatment, 43.4% for observation. Recurrence rates were 2.6% and 13.8%, respectively (*P* = 0.106). Overall recurrence rate was 5.5%. *Conclusions.* HNBCC commonly presented as pigmented ulcers over the nose of elderly female patients in our locality. Adequate tumour excision ± reconstruction offered the best chance of cure. Reexcision of those with inadequate skin margins improved local tumour control.

## 1. Introduction

The incidence of skin cancer is increasing worldwide, possibly arising from an ageing population and increasing sunlight exposure. Basal cell carcinoma (BCC) is the most prevalent. The incidence of which is increasing at 3% per annum [1–7]. 

BCC most often arise in areas of long-term sun exposure with a high predilection for the head and neck area. Classical clinical features include raised and rolled edges, pearly central area with telangiectasia. Lesions may be pigmented or nonpigmented; nodular, ulcerative, erythematous patches or even mimic benign lesions. Most BCCs are indolent with a low incidence of metastasis [8–11]. 

The mainstay of treatment for BCC of the head and neck region (HNBCC) is surgical excision with adequate margins. Radiotherapy may be advocated as definitive treatment for a selected group of patients, for those unfit for surgery or as an adjuvant treatment for those with inadequate margins. Other treatment modalities such as curettage and electrodessication, cryosurgery have been reported with variable treatment outcomes; similarly, topical and intralesional agents, photodynamic therapy have also been described, again with variable outcomes, warranting careful patient selection [11–14]. 

Much has been published regarding BCCs in Caucasian populations, but data on ethnic Chinese are less readily available. We herein report the patient demographics, tumour characteristics, surgical management, and outcome of HNBCCs in ethnic Chinese patients in Hong Kong.

## 2. Materials and Methods

A retrospective review of all ethnic Chinese patients with previously untreated HNBCC managed in the Queen Mary Hospital, The University of Hong Kong, Hong Kong, between 1999 and 2009. Queen Mary Hospital was one of the largest regional hospitals in Hong Kong. It was a tertiary and quaternary referral centre for the entire territory. Outcome measures included patient demographics, tumour characteristics, surgical management, management of patients with inadequate margins, and recurrence rates. Statistical analysis was performed using SPSS 18.0.

## 3. Results

### 3.1. Patient Demographics

A total of 226 HNBCC patients of Chinese ethnicity were treated in our centre from 1999 to 2009. Mean age was 73.1 (22–100) years. There were 132 female and 94 male patients with a male to female ratio of 0.7. 25 patients had multiple BCC lesions. There were a total of 273 HNBCC lesions. 

### 3.2. Tumour Characteristics

**Figure 1:**
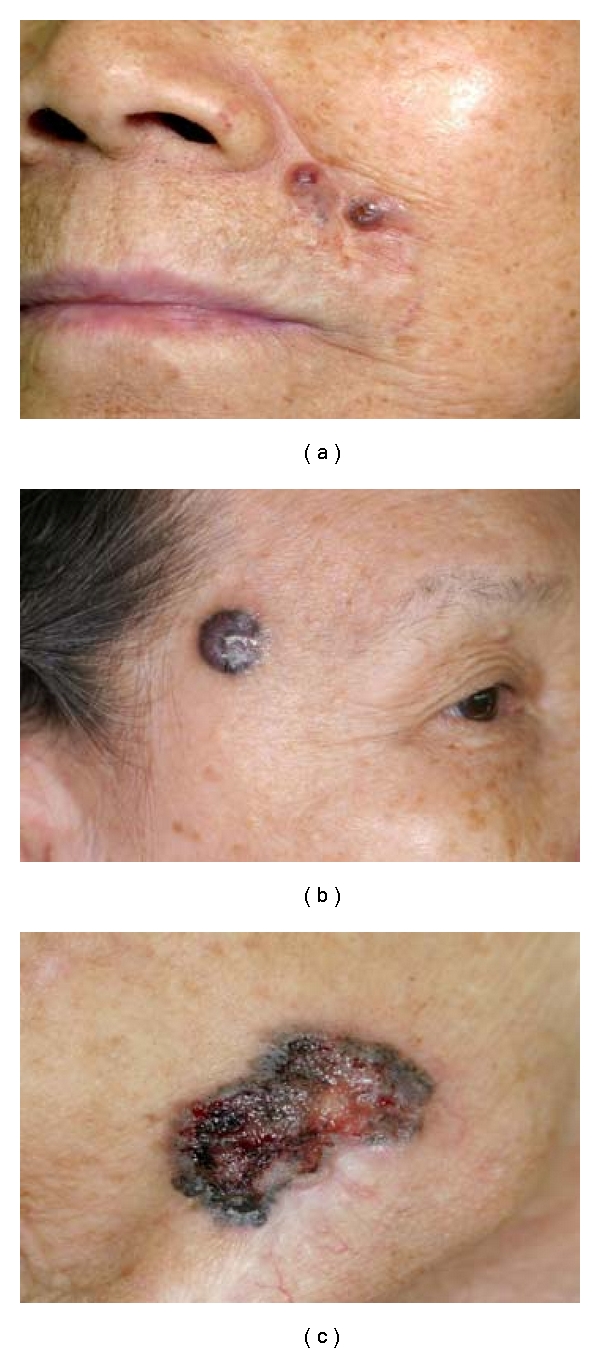
Common presentation of HNBCC in ethnic Chinese: pigmented lesion with well-defined borders, rolled ulcer edges, central pearly area, and overlying telangiectasia.

**Figure 2:**
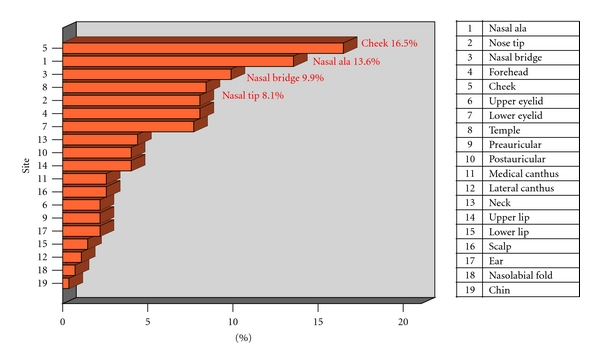
HNBCC presentation—anatomical sites. The commonest site was on the nose.

There were 65 (23.8%) nonpigmented and 208 (76.2%) pigmented lesions. The most common presentation was in the form of an ulcer (64.8%, *n* = 177), followed by nodule (19.3%, *n* = 53), erythema (1.1%, *n* = 3), and lesions that mimic a benign lesion, for example, keratosis (14.7%, *n* = 40) (Figure 1). Common sites of involvement included the nose (31.6%, *n* = 86) and cheek (16.5%, *n* = 45) (Figure 2). 

### 3.3. Management

**Figure 3:**
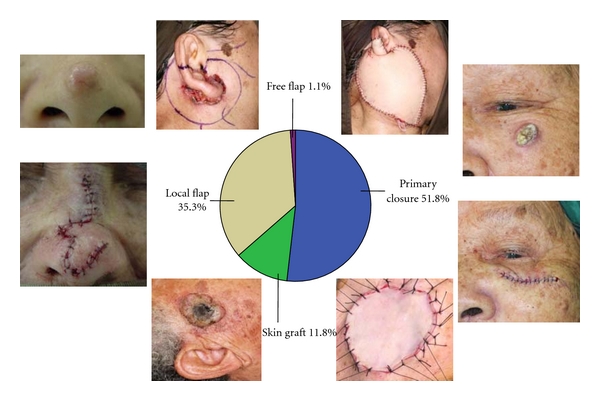
Methods of wound closure. From right to left in a clockwise direction: an 80-year-old lady with a pigmented ulcerative BCC over her left cheek, pathology excised with a 2 mm margins, wound closed primarily; a 60-year-old lady with a pigmented ulcerative BCC over her right preauricular region, the wound was too extensive for primary closure after tumour resection, and yet there was insufficient tissue for local flap reconstruction, hence a full thickness skin graft was harvested from the postauricular region for wound coverage; a 70-year-old lady with a nonpigmented nodular BCC over her nose tip. Excision was performed with a 2 mm margin followed by reconstruction with a bilobed flap; a 50-year-old lady who presented with a pigmented ulcerative BCC over her right auricle which invaded into the superficial and deep lobes of the parotid gland; facial nerve was intact. Wide local excision of tumour with total conservative parotidectomy was performed. The defect was reconstructed with a free anterolateral thigh myocutaneous flap.

One patient with solitary HNBCC refused treatment. All others underwent surgical excision. Median skin margin taken on tumour excision was 2.0 (0–20) mm. Primary skin closure was achieved in 51.8% (*n* = 141). Other patients required reconstruction in the form of skin graft (11.7%, *n* = 32), local flap (35.3%, *n* = 96), and free flap (1.1%, *n* = 3) (Figure 3).

### 3.4. Treatment Outcomes

**Figure 4:**
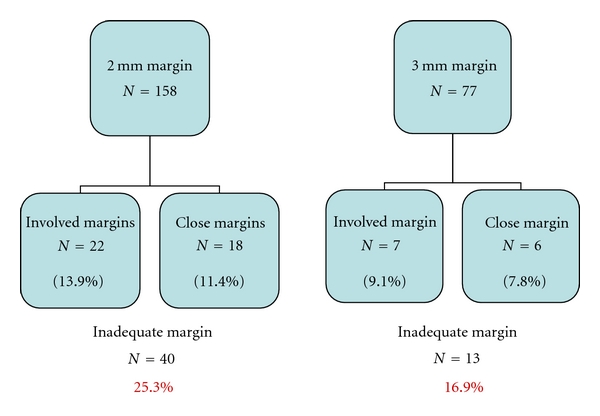
Treatment outcome—the greater the skin margin taken on tumour excision, the better the tumour clearance rate.

Skin margins were uninvolved, involved, and close in 75.4% (*n* = 205), 15.4% (*n* = 42), and 9.2% (*n* = 25), respectively, with close skin margin being defined as a pathological margin of less than one millimeter. Involved and close skin margins were classified as inadequate skin margins. The rate of tumour clearance was increased with an increase in skin margin taken on tumour excision—25.3% (*n* = 40) inadequate margins for 2 mm margins versus 16.9% (*n* = 13) for 3 mm margins (*P* = 0.089) (Figure 4). 

**Figure 5:**
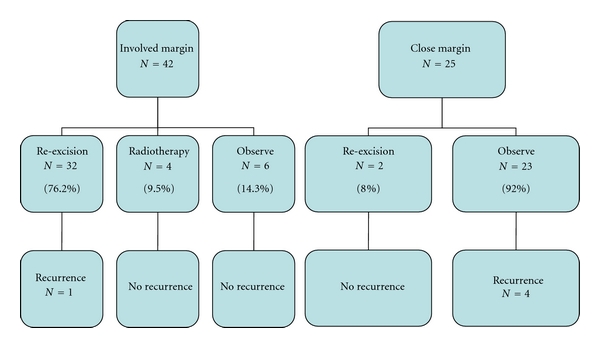
Management of patients with involved and close margins and associated recurrence rates.

Most patients with involved margins (76.2%, *n* = 32) underwent reexcision; 4 (9.5%) underwent radiation therapy. One developed local recurrence 2 years after reexcision. The majority of those with close margins (92.0%, *n* = 23) opted for observation. Four developed local recurrence thereafter. Two patients opted for reexcision, and none recurred to date (Figure 5). 

**Figure 6:**
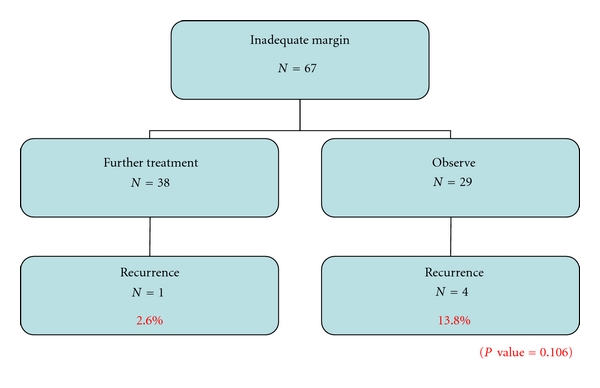
Treatment outcomes of patients who chose to undergo further treatment versus observation in those with inadequate margins.

**Figure 7:**
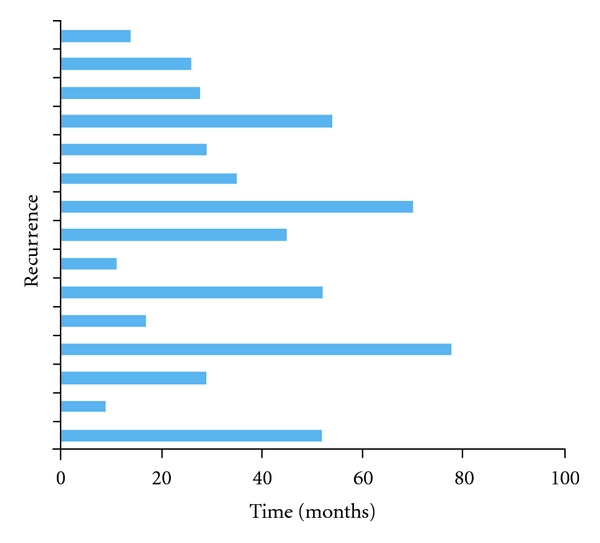
A graph depicting tumour recurrence over time.

Further treatment in the form of reexcision or radiation therapy in those with inadequate skin margins led to a lower recurrence rate than those who opted for observation (2.6% versus 13.8%, *P* = 0.106) (Figure 6). Follow-up period was indefinite for all patients. Overall recurrence rate was 5.5% (*n* = 15) over a mean follow-up period of 73.0 (16–195) months. Mean interval to recurrence was 36.6 (9–78) months (Figure 7). The commonest site of recurrence was over the nose 20% (*n* = 3). 

## 4. Discussion

According to the Hong Kong Hospital Authority statistical report 2007-2008, there was an increasing trend of skin cancer in Hong Kong [15]. The rate of increase in skin cancer and BCC incidence in ethnic Chinese and other Asian countries was less than that of the fair-skinned Caucasian population [16]. 

BCC had a high predilection for the head and neck region. HNBCC predominantly affected the elderly population with a slight female preponderance in our locality. Common presentation was in the form of a pigmented ulcer over the nose. 

Our results were similar to those reported by Sng et al., Kikuchi et al., and Cho et al. [6, 17, 18]. This study, corroborated by reviews in other Asian countries, showed that HNBCC in ethnic Chinese and other Asian populations presented differently compared to the Caucasian population, whereby HNBCC commonly presented as nonpigmented nodules in male patients (Table 1) [6, 11, 17–19]. 

The difference in trends, rates, and presentation in the two ethnic groups could be accounted for the difference in skin types (Fitzpatrick types III and IV in Chinese versus I and II in Caucasians), geographical latitude, sociocultural differences, varying occupational and sun exposure, skin protection, and differences in disease awareness and surveillance. 

There is no consensus as to the amount of skin margin taken on tumour excision. However, as one would expect, the greater the skin margin taken on tumour excision, the better the tumour control. In excising tumour over the head and neck region, there is always a balance between adequate tumour control and conservation of normal tissue in an attempt to achieve acceptable functional and cosmetic outcome. Excision of HNBCC with a 2 mm skin margin for well-defined lesions was adequate in most cases, with an overall recurrence rate of 5.5%, which was comparable to that of large-scale studies conducted worldwide [20–23]. 

As cited in other reviews, our data also showed that the nose was the site with the highest incidence of recurrence and inadequate margins. This could represent embryonic fusion planes where tumour can spread aggressively or because of a scarcity of surplus skin tissue which may present technical difficulty on skin closure, resulting in a more conservative excision margin [24–27]. 

For such difficult mid-face lesions, excision under frozen section guidance or even Mohs micrographic surgery could be advocated to enhance complete tumour removal whilst preserving the maximal amount of normal tissue [11, 19, 23, 28–30]. Various reconstructive techniques such as skin graft, local flaps, or even free flaps could be used for skin coverage if the defect is too extensive for primary closure. 

In cases of inadequate skin margins, reexcision should be advocated to prevent recurrence and to decrease the chance of more radical surgery in the future; incompletely excised tumour and recurrent tumours are contributing factors to more aggressive tumour behaviour. The presence of scar tissue obscures monitoring and delays clinical detection. Fibrotic scar tissue entraps malignant cells and favours deep extension by preventing upward migration [27, 31, 32]. 

## 5. Conclusions

HNBCC commonly presented as pigmented ulcers over the cheek and nose of elderly female patients of Chinese ethnicity in our locality. This corroborated with studies conducted in other Asian countries but contrasted with those of Caucasian populations in that HNBCC commonly presented as nonpigmented nodules in the male population. Tumour excision with a 2 mm skin margin for HNBCC with well-defined margins yielded a tumour control rate comparable with other large-scale studies. Various reconstructive techniques could be adapted in cases where primary skin closure could not be achieved after adequate tumour resection. Reexcision of lesions with inadequate margins improved local tumour control.

##  Conflict of Interests

The authors declare that there is no conflict of interests.

## Figures and Tables

**Table 1:** A table comparing our results with other Asian and Caucasian populations.

	Hong Kong (QMH) *N* = 273	Singapore *N* = 292	Japan *N* = 243	Korea *N* = 78	Australia *N* = 6252
Mean age (yrs)	73.1	70.9	59.0	58.2	62.0
M:F	0.70	0.95	0.97	0.90	1.13
Clinical features	Pigmented (76.2%)	Pigmented (63%)	Pigmented (75%)	Pigmented (55%)	Nonpigmented (93%)
	Ulcer (64.8%)	Ulcer (NA)	Nodule (NA)	Ulcer (NA)	Nodule (50%)
Site	Nose (32.3%)	Nose (37.0%)	Nose (NA)	Nose (26.9%)	Nose (40.6%)
